# Progressive Changes in Sleep and Its Relations to Amyloid-β Distribution and Learning in Single *App* Knock-In Mice

**DOI:** 10.1523/ENEURO.0093-20.2020

**Published:** 2020-04-29

**Authors:** Sakura Eri B. Maezono, Mika Kanuka, Chika Tatsuzawa, Miho Morita, Taizo Kawano, Mitsuaki Kashiwagi, Pimpimon Nondhalee, Masanori Sakaguchi, Takashi Saito, Takaomi C. Saido, Yu Hayashi

**Affiliations:** 1 International Institute for Integrative Sleep Medicine (WPI-IIIS), University of Tsukuba, Tsukuba, Ibaraki 305-8575, Japan; 2 PhD Program in Human Biology, School of Integrative and Global Majors, University of Tsukuba, Tsukuba, Ibaraki 305-8575, Japan; 3 Doctoral Programs in Kansei, University of Tsukuba, Tsukuba, Ibaraki 305-8575, Japan; 4 Laboratory for Proteolytic Neuroscience, RIKEN Center for Brain Science, Wako, Saitama 351-0198, Japan; 5 Department of Neurocognitive Science, Institute of Brain Science, Nagoya City University Graduate School of Medical Science, Nagoya, Aichi 467-8601, Japan

**Keywords:** Alzheimer’s disease, brainstem, electroencephalogram, learning and memory, mouse, sleep

## Abstract

Alzheimer’s disease (AD) patients often suffer from sleep disturbances. Alterations in sleep, especially rapid eye movement sleep (REMS), can precede the onset of dementia. To accurately characterize the sleep impairments accompanying AD and their underlying mechanisms using animal models, it is crucial to use models in which brain areas are affected in a manner similar to that observed in the actual patients. Here, we focused on *App^NL-G-F^* mice, in which expression levels and patterns of mutated amyloid precursor protein (APP) follow the endogenous patterns. We characterized the sleep architecture of male *App^NL-G-F^* homozygous and heterozygous mice at two ages (six and 12 months). At six months, homozygous mice exhibited reduced REMS, which was further reduced at 12 months together with a slight reduction in non-REMS (NREMS). By contrast, heterozygous mice exhibited an overall normal sleep architecture. Homozygous mice also exhibited decreased electroencephalogram γ to δ power ratio during REMS from six months, resembling the electroencephalogram slowing phenomenon observed in preclinical or early stages of AD. In addition, homozygous mice showed learning and memory impairments in the trace fear conditioning (FC) at both ages, and task performance strongly correlated with REMS amount at 12 months. Finally, histologic analyses revealed that amyloid-β accumulation in the pontine tegmental area and ventral medulla followed a course similar to that of the REMS reduction. These findings support the notion that changes in REMS are an early marker of AD and provide a starting point to address the mechanism of sleep deficits in AD and the effects on cognition.

## Significance Statement

Sleep impairments are major non-cognitive symptoms in Alzheimer’s disease (AD). Moreover, recent animal studies provide strong support that insufficient sleep accelerates neurodegeneration. Towards elucidating how and why sleep is impaired in AD using animal models, minimizing artefacts due to overexpressing or ectopically expressing *App* is crucial. Here, we addressed the effects of singly knocking in the human *App* gene carrying mutations associated with familial AD into the mouse *App* locus. The mutations lead to progressive changes in rapid eye movement sleep (REMS), which correlated with memory deficits and amyloid-β distribution in the brainstem, thus pointing out the importance of studying REMS in understanding AD-associated sleep impairments. In addition, EEG alterations during REMS appeared earlier than during wake, which might be beneficial as biomarkers.

## Introduction

Alzheimer’s disease (AD) is a slowly progressing neurodegenerative disease characterized by extracellular amyloid-β (Aβ) deposits, intracellular neurofibrillary tangles, and neuronal loss. In addition to cognitive impairments, sleep disturbances commonly occur in patients with AD (Carpenter et al., 1996; McCurry et al., 1999). Sleep impairments can exacerbate a decline in the quality of life, not only of the patients with AD, but also that of the caregivers (Moran et al., 2005). Moreover, recent studies in humans and animal models revealed that sleep deprivation or fragmentation accelerates Aβ accumulation and may thus contribute to the progression of AD (Kang et al., 2009; Minakawa et al., 2017; Shokri-Kojori et al., 2018). This notion is further supported by recent findings that alterations in sleep are present before the onset of AD (Ju et al., 2013; Pase et al., 2017).

In attempts to characterize the sleep disturbances accompanying AD and to elucidate the underlying mechanisms, many studies have conducted sleep recordings in various mouse models of AD (Jyoti et al., 2010; Platt et al., 2011; Roh et al., 2012; Schneider et al., 2014; Colby-Milley et al., 2015; Sethi et al., 2015; Kent et al., 2018). The mouse models used in these studies, however, either carry multiple copies of *App* or *presenilin* or use heterologous promoters to express these genes, which likely leads to overexpression or ectopic expression of amyloid precursor protein (APP) or presenilin, factors that contribute to the generation of Aβ from APP. The phenotypes of such AD mouse models may be due in part to an unintended consequence of the overexpression. Moreover, sleep/wake states are regulated by the interactions of various brain areas, and ectopic expression of APP or presenilin may affect such interactions and alter sleep in a largely different manner than in patients with the actual disease.

To overcome these concerns, we focused on the *App^NL-G-F^* mouse, a recently developed mouse model of AD in which a mutated human version of *App* is singly knocked into the original *App* locus (Saito et al., 2014). In these mice, the humanised *App* sequence contains three mutations that are associated with familial AD: the Swedish (NL), Beyreuther/Iberian (F), and Arctic (G) mutations. These mice do not exhibit elevated expression of APP, but do exhibit a progressive increase in the accumulation of Aβ, a higher ratio of Aβ42 to Aβ40, amyloidosis, and neuroinflammation in several brain areas (Saito et al., 2014).

To evaluate how the sleep architecture and state-dependent oscillatory brain activities are affected in *App^NL-G-F^* heterozygous and homozygous mice, we recorded the EEG and EMG from these mice at multiple ages. Furthermore, to gain insight into the brain areas responsible for the altered sleep patterns, amyloidosis was assessed in several subcortical areas involved in sleep regulation. In addition, to investigate the relationship between the development of sleep disturbances and cognitive impairment, we assessed the learning and memory abilities in these mice and analyzed the correlation between their performance in the behavioral tasks and sleep parameters.

## Materials and Methods

### Animals

Male and female *App^NL-G-F/wt^* mice on a C57BL/6J background were crossed to obtain male *App^NL-G-F/wt^*, *App^NL-G-F/NL-G-F^*, and control wild-type (WT) mice for analyses. The mice were group housed under a 12/12 h light/dark cycle (lights on at 9 A.M.) under controlled temperature (23.5 ± 2.0°C) and humidity conditions (51.0 ± 10.0%) with free access to water and food. The mouse facility was SPF grade, and solid plastic cages (CLEA Japan) and paper chip bedding (Sankyo Labo Service Corp.) were used. All animal experiments were approved by the Institutional Animal Care and Use Committee of the University of Tsukuba, and all procedures were conducted in accordance with the Regulations for Animal Experiments of the University of Tsukuba.

### Behavioral tests

Male mice underwent the open field test (OFT) and trace fear conditioning (FC) test, which are described in detail below. Before each behavioral test, the mice were each handled for 4 d (2 min × two times on the first day and 2 min × three times for the next 3 d) according to a previous study (Purple et al., 2017). The orders in which mice of different genotypes underwent behavioral experiments were randomized. During the experimental procedures and subsequent data analyses, the experimenter was blinded to the genotype.

### OFT

The OFT was performed as described in a previous study (Seibenhener and Wooten, 2015) with some modifications. Briefly, mice were individually placed in an acrylic box (40 × 40 × 40 cm) and the activity was monitored by a video camera positioned centrally above the box. Each session lasted 10 min per mouse and was performed between zeitgeber time (ZT) 3:00 and 5:00. Light intensity was fixed at 70 lx and white noise (80 dB) was applied. Video files were analyzed using the SMART Video Tracking software v3 (PanLab/Harvard Apparatus). The open field was divided into 16 equivalent square areas and the four inner squares were considered the central zone.

### Trace FC

The trace FC test was performed as previously described (Chowdhury et al., 2005; Purple et al., 2017) with some modifications. On day 1, mice were trained between ZT 8:30 and 9:30. The training context (context A) was a chamber (31 × 24 × 21 cm) equipped with a stainless steel shock grid floor, as previously described (Arruda-Carvalho et al., 2011). After 192 s in context A, each mouse received five sets of conditioned stimulus (CS)-unconditioned stimulus (US) pairs. The CS was a 20-s tone (∼80 dB) and the US was a 2-s foot shock (0.75 mA). The CS and US were separated by a 10-s trace period. Between each set, there was a 180-s interval. Mice remained in the same context for an additional 180 s before being returned to their home cage. On day 2, a retrieval test was performed between ZT 6:00 and 8:00. Mice were placed in a novel environment (context B), which was an acrylic box (570 × 370 × 185 cm) wrapped outside with black paper sheets. After 192 s in context B, the CS was presented. During both the training and the retrieval test, mouse activity was monitored by a video camera to calculate the freezing rate. On day 1, the responsivity to the shock stimulus was assessed as described in our previous study (Purple et al., 2017) with some modifications. Briefly, the distance of the mouse movement 2 s before and during the first shock presentation was measured using Freezeframe4 (Actimetrics Software), which digitized the video signal at ∼10 Hz and allowed for measurement of the movement frame by frame. Mice were judged as exhibiting freezing behavior if no movement except for that related to respiration was detected for at least 1 s by an experimenter blinded to the genotype.

### EEG/EMG recording and analyses

Male mice were subjected to EEG/EMG recording to characterize the sleep architecture. To implant EEG and EMG electrodes, the mice were anaesthetized with isoflurane and placed in a stereotaxic frame (Leica Angle Two, Leica Microsystems Inc.). Core body temperature was maintained using a feedback-controlled heating pad. EEG electrodes were stainless steel recording screws implanted epidurally over the parietal cortex (3 mm posterior to bregma, 1.5 mm lateral to the midline) and cerebellum (6.5 mm posterior to bregma, 2 mm lateral to the midline). EMG electrodes were stainless steel Teflon-coated wires placed bilaterally into nuchal muscles. The electrodes were fixed to the skull with the resin cement (Super-Bond C&B set; Sun Medical). The mice were allowed to recover in their home cages for two weeks before being transferred to the sleep recording chambers. The mice were attached to the recording cables and acclimatized to the recording chamber for at least 5 d. Following 48 h of EEG/EMG recording under basal conditions, novel objects (marbles) were presented and EEG/EMG was recorded for an additional 4 h. The EEG/EMG data were amplified and filtered (band pass 0.5–250 Hz), digitized at a sampling rate of 512 Hz, and collected using VitalRecorder (Kissei Comtec).

The EEG signals were subjected to fast Fourier transform and further analysis using SleepSign (Kissei Comtec). The vigilance state in each epoch was manually classified as rapid eye movement sleep (REMS), non-REMS (NREMS), or wakefulness, on the basis of the EEG patterns as well as the absolute δ (1–4 Hz) power, the θ (7–10 Hz) power to δ power ratio, and the integral of the EMG signals. Epochs with high EMG and low δ power were classified as wakefulness. Epochs with high δ power and low EMG were classified as NREMS. Epochs with even lower EMG (suggestive of muscle atonia) and a high θ power to δ power ratio were classified as REMS. If a single epoch contained multiple states, the state with the highest occupancy was assigned. The epochs were 4 s long. In calculating the EEG power spectrum of REMS, NREMS, or wakefulness, the EEG power of each frequency bin was expressed as a percentage of the mean total EEG power over all frequency bins (1–50 Hz) across 24 h. All manual scoring was performed by an experimenter blinded to the genotype.

### Immunohistochemistry

Following transcardial perfusion with 0.1 m PBS, dissected brains were postfixed with 4% paraformaldehyde/PBS overnight, equilibrated with 30% sucrose/PBS, and sectioned at 40 μm using a microtome (Yamato Kohki). The sections were washed with distilled water and placed in 0.3% H_2_O_2_/MeOH for 30 min and washed with distilled water again. After washing with 1× TBS (pH 7.5) + 0.1% Tween 20 (TBST) and incubating for 30 min in Tris-NaCl-blocking buffer (1× TBS + 0.5% Blocking Reagent; PerkinElmer; FP1020), the sections were incubated with a primary antibody for choline acetyltransferase (ChAT; 1:100 goat anti-ChAT; EMD Millipore; AB144P) at room temperature overnight and washed three times with TBST. The sections were then incubated with a primary antibody for Aβ [1:1000 mouse anti-human Aβ (N; 82E1) IgG; IBL; 10 323] at 4°C overnight. The sections were washed three times with TBST and incubated with secondary antibodies for ChAT [1:500 donkey anti-goat IgG-Alexa Fluor 546; Invitrogen; A-11056] and Aβ [1:1000 donkey anti-mouse horseradish peroxidase; Abcam; ab7061] combined with 1 μg/ml 4’,6-diamidino-2-phenylindole for 120 min. After washing four times in TBST, the sections were incubated with fluorescein-tyramide reagent (PerkinElmer; SAT701001KT) for 30 min and then washed four times in TBST. All sections were mounted on a slide glass using Immu-Mount (Thermo Scientific Shandon; 9990412). Images of the brain sections were obtained with a digital slide scanner (NanoZoomer XR, Hamamatsu Photonics). Quantification of Aβ-derived signals was performed as described in a previous study (DeVos et al., 2018) with some modifications. Briefly, the regions of interest were set manually using a freehand selection tool by an experimenter blinded to the genotype. Images were then processed using an ImageJ-based algorithm (DeVos et al., 2018) with some modifications. For each individual mouse, the calculated mean plaque area from, typically, three to four coronal sections (at least two sections) was considered the plaque area for each brain region of that mouse. For the medial septum-diagonal band of Broca (MSDB), the ChAT-positive area was chosen within this region from coronal sections between bregma 1.10 mm and 0.62 mm. For the pons, the tegmental area dorsal to the motor trigeminal nucleus and ventral to the cuneiform nucleus in coronal slices between bregma −4.84 and −5.34 mm was selected. For the medulla, the area ventromedial to either the facial nucleus or the ambiguous nucleus in coronal slices between bregma −6.34 and −6.84 mm was selected.

### Experimental design and statistical analyses

The experimenters performing the data analyses were blinded to the genotype when scoring or analysing EEG/EMG, behavioral, or histologic data. Statistical analyses were performed using SPSS (IBM Corp.), PRISM 8 (GraphPad Software), or R statistical software (http://www.r-project.org/). Bar graphs and line graphs represent mean ± standard error of the mean (SEM). Each point on the bar graphs and scatter plots represents an individual mouse. For comparisons among three groups with multiple timepoints/trials, mixed ANOVA and *post hoc* Games–Howell multiple comparison test were applied. Otherwise, Games–Howell multiple comparison test or Welch’s *t* test was applied for comparisons among three or two groups, respectively. For correlation analyses, Pearson’s correlation coefficient (*r*) and the *p* value were calculated. Where applicable, all statistical tests were two-tailed. Significance was set at *p *<* *0.05. Details on sample sizes and results of statistical tests are described in Extended Data.

## Results

To assess the sleep architecture, learning abilities, and Aβ plaque distribution in *App^NL-G-F/wt^* and *App^NL-G-F/NL-G-F^* mice at multiple ages, two independent mouse groups were subjected to experiments with different timelines. For each of the two groups, sleep recording was performed at six months or 12 months of age. After the sleep recording, trace FC was performed at seven or 13 months of age. When mice were further subjected to histologic analyses, the mice were immediately killed by an overdose of anesthesia following trace FC to avoid any long-term effects of the fear experience.

**Figure 1.**
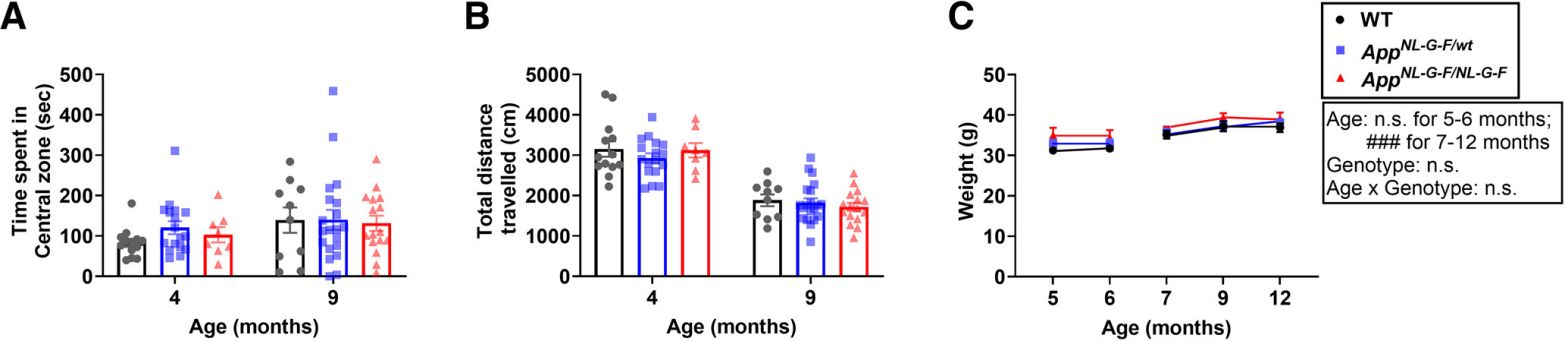
Normal OFT performance and body weight in *App^NL-G-F/wt^* and *App^NL-G-F/NL-G-F^* mice. ***A***, ***B***, Time spent in the central zone (***A***) and total distance traveled (***B***) by four- and nine-month-old mice. Bar graphs represent mean ± SEM. Each point represents an individual mouse. ***C***, The body weight of the younger mice was measured at five and six months while the body weight of the older mice was measured at seven, nine, and 12 months. Each point represents the mean ± SEM. Detailed results of the statistical tests are described in Extended Data Figure 1-1.

In addition, OFT was performed to evaluate anxiety and locomotor activity at four or nine months (Fig. 1*A*,*B*). At neither age did heterozygous or homozygous mice display overt anxiety-like behavior, as reflected by a decrease in the time spent in the central zone (Fig. 1*A*), or decreased locomotion according to the total distance traveled (Fig. 1*B*). The body weight of heterozygous and homozygous mice also appeared equivalent to that of WT controls at all tested ages (Fig. 1*C*).

10.1523/ENEURO.0093-20.2020.f1-1 Extended Data Figure 1-1 Detailed results of the statistical analyses in Figure 1. Download Figure 1-1, XLSX file.

### Progressive deterioration of sleep architecture in single *App* knock-in mice

**Figure 2.**
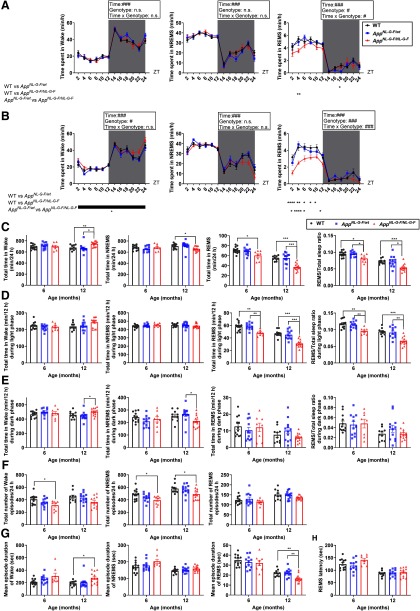
*App^NL-G-F/NL-G-F^* mice exhibit age-dependent impairments in sleep architecture. ***A***, ***B***, Diurnal sleep-wake cycles of six-month-old (***A***) and 12-month-old (***B***) mice. Each point represents the mean ± SEM; #*p *<* *0.05, ###*p *<* *0.001, mixed ANOVA; *p <* *0.05, **p <* *0.01, ***p <* *0.001, ****p < 0.0001, *post hoc* Games–Howell multiple comparison test. ***C–E***, Total amount of wake, NREMS, REMS, and ratio of REMS to total sleep [24 h (***C***), light period (***D***), and dark period (***E***)]. ***F***, ***G***, Number of episodes (***F***) and mean episode duration (***G***) in each stage of wake, NREMS, and REMS. ***H***, REMS latency. Bar graphs represent the mean ± SEM. Each point represents an individual mouse; *p <* *0.05, **p <* *0.01, ***p <* *0.001, Games–Howell multiple comparison test. Detailed results of the statistical tests are described in Extended Data Figure 2-1.

The sleep architecture of *App^NL-G-F/wt^*, *App^NL-G-F/NL-G-F^*, and control WT mice was compared using 24-h recordings of EEG and EMG at either six or 12 months of age (Fig. 2). At six months, a reduction of the total time in REMS and in the REMS/total sleep ratio was detected in homozygous mice (Fig. 2*C*). This reduction in the total time spent in REMS was mostly attributed to changes in the light phase (resting phase; Fig. 2*A*,*D*,*E*). A decrease in the number of wake and NREMS episodes was also observed (Fig. 2*F*). At 12 months, a further reduction in the total time spent in REMS and the REMS/total sleep ratio was detected in the homozygous mice (Fig. 2*C*), together with a shorter mean episode duration (Fig. 2*G*). Again, the reduction in the total time spent in REMS was mostly attributed to changes in the light phase (Fig. 2*B*,*D*,*E*). In addition, a decrease in the total time spent in NREMS and an increase in the total time spent awake was observed at this age (Fig. 2*C*). In contrast to REMS, the change in the amount of wake and NREMS seemed to arise from changes in the dark phase (active phase; Fig. 2*D*,*E*). For the wake state, the mean episode duration was increased, whereas for NREMS, the number of episodes was decreased (Fig. 2*F*,*G*). REMS latency was not affected at six or 12 months (Fig. 2*H*). The sleep architecture of heterozygous mice was grossly similar to that of the age-matched WT control mice at both six and 12 months (Fig. 2).

10.1523/ENEURO.0093-20.2020.f2-1 Extended Data Figure 2-1 Detailed results of the statistical analyses in Figure 2. Download Figure 2-1, XLSX file.

### Alterations of brain oscillatory activities during sleep in single *App* knock-in mice

**Figure 3.**
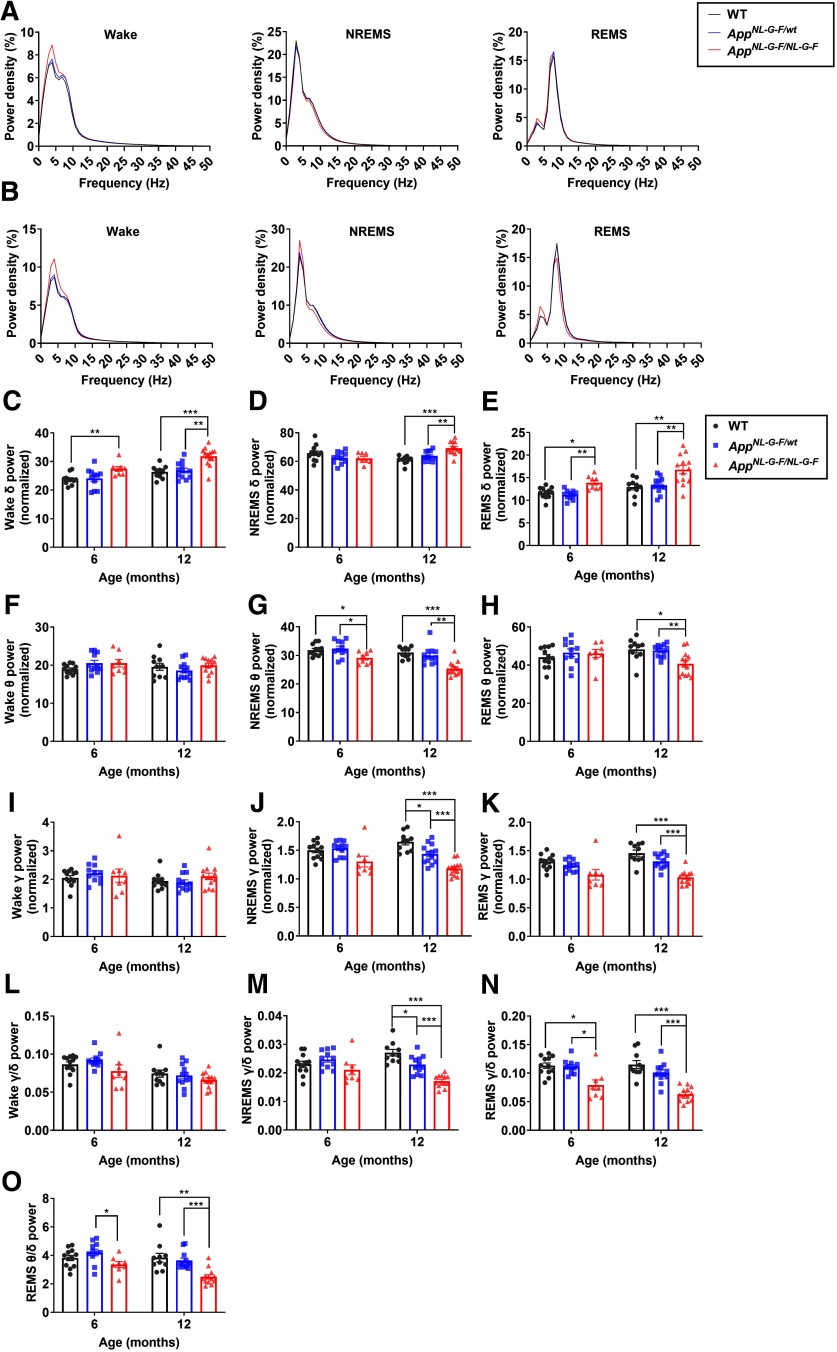
*App^NL-G-F/NL-G-F^* mice exhibit age-dependent alterations in brain oscillatory activities. ***A***, ***B***, EEG power spectrum of wakefulness, NREMS, and REMS in six-month-old (***A***) and 12-month-old (***B***) mice. ***C–K***, Comparison of δ (1–4 Hz) power (***C–E***), θ (7–10 Hz) power (***F–H***), and γ (25–45 Hz) power (***I–K***) during wake (***C***, ***F***, ***I***), NREMS (***D***, ***G***, ***J***), and REMS (***E***, ***H***, ***K***). ***L–N***, Ratio of γ to δ power during wake (***L***), NREMS (***M***), and REMS (***N***). ***O***, Ratio of θ to δ power during REMS. Bar graphs represent mean ± SEM. Each point represents an individual mouse; *p <* *0.05, **p <* *0.01, ***p <* *0.001, Games–Howell multiple comparison test. Detailed results of the statistical tests are described in Extended Data Figure 3-1.

The brain exhibits oscillatory activities across various frequencies with distinct patterns depending on the vigilance state. Such oscillatory activities are thought to play important roles in information processing and are altered in many neuronal diseases, including AD, with distinct characteristics depending on the disease (Herrmann and Demiralp, 2005; Koenig et al., 2005). In AD, alterations in the oscillatory activities are most readily detected during REM sleep, with a decrease in high-frequency oscillations accompanied by an increase in low-frequency oscillations (Prinz et al., 1992; Petit et al., 1993). These alterations are also detected in subjects with mild cognitive impairment, suggesting that the alterations emerge from the preclinical stage of AD (Brayet et al., 2016). To investigate whether brain oscillatory changes occur in *App^NL-G-F/wt^* and *App^NL-G-F/NL-G-F^* mice, the power spectra of EEG obtained at different vigilance states were compared between genotypes at six and 12 months (Fig. 3). At six months, the homozygous mice had a significantly higher δ power during REMS compared with WT, whereas the δ power during NREMS was not affected (Fig. 3*D*,*E*). By contrast, during NREMS, θ power was significantly lower in the homozygous mice (Fig. 3*G*). For both sleep stages at six months, although not significant, there was a trend toward decreased γ power in the homozygous mice (Fig. 3*J*,*K*). At 12 months, the oscillatory activity during sleep was further affected in the homozygous mice. During both sleep stages, δ power was significantly increased, whereas θ and γ power were significantly decreased (Fig. 3*D*,*E*,*G*,*H*,*J*,*K*). Oscillatory activity during the wake state was less affected in the homozygous mice. θ And γ power appeared normal at both ages, whereas δ power was increased (Fig. 3*C*,*F*,*I*).

10.1523/ENEURO.0093-20.2020.f3-1 Extended Data Figure 3-1 Detailed results of the statistical analyses in Figure 3. Download Figure 3-1, XLSX file.

Considering the reported decrease in high-frequency oscillations and the increase in low-frequency oscillations in patients with early AD, we next compared the ratio of fast oscillatory (γ) power to slow oscillatory (δ) power. The ratio was significantly lower during REMS in the homozygous mice at six months, when the ratio appeared unaffected during wake or NREMS (Fig. 3*L*,*M*,*N*). In addition, the ratio of θ power to δ power during REMS was reduced in the homozygous mice (Fig. 3*O*).

### Sleep under novel object presentation

**Figure 4.**
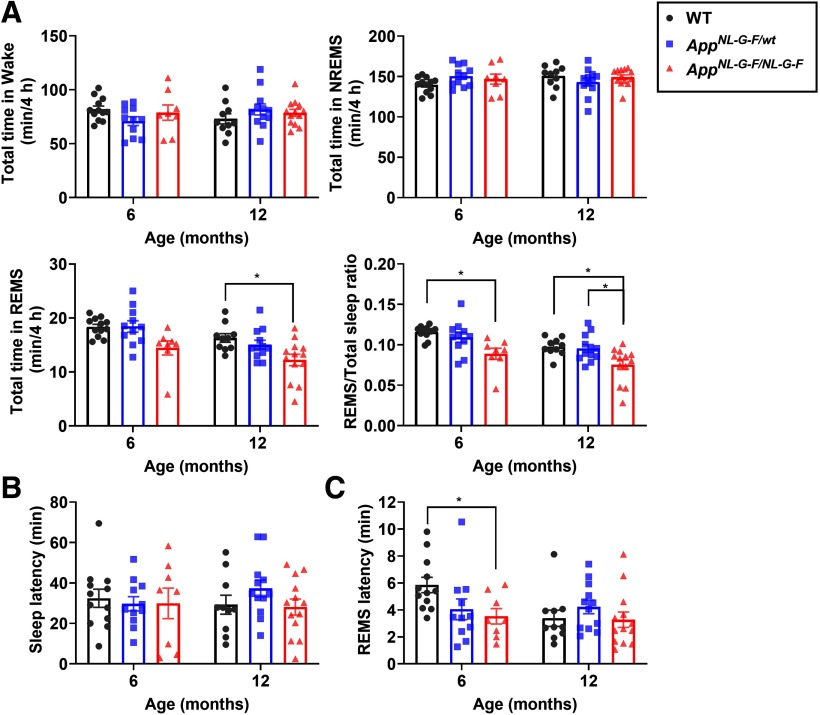
*App^NL-G-F/NL-G-F^* mice exhibit reduced REMS following exposure to novel objects. ***A***, Total amount of wake, NREMS, REMS, and ratio of REMS to total sleep following presentation of novel objects. ***B***, ***C***, Latency to sleep (***B***) or REMS (***C***) following presentation of novel objects; *p <* *0.05, Games–Howell multiple comparison test. Bar graphs represent mean ± SEM. Each point represents an individual mouse. Detailed results of the statistical tests are described in Extended Data Figure 4-1.

Human sleep is often affected by external stimuli. Exposing rodents to novel objects also affect their sleep (Schiffelholz and Aldenhoff, 2002). Therefore, we next examined how the presentation of novel objects affected sleep in *App^NL-G-F/wt^* and *App^NL-G-F/NL-G-F^* mice. Sleep was recorded from these mice and WT control mice for 4 h on exposure to novel objects at six and 12 months (Fig. 4). At both ages, similar to undisturbed sleep, the REMS/total sleep ratio was reduced in the homozygous mice (Fig. 4*A*). In addition, the REMS latency was decreased in the homozygous mice at six months (Fig. 4*C*). Thus, under specific conditions, the first episode of NREMS was shortened in these mice.

10.1523/ENEURO.0093-20.2020.f4-1 Extended Data Figure 4-1 Detailed results of the statistical analyses in Figure 4. Download Figure 4-1, XLSX file.

### Learning and memory impairment in single *App* knock-in mice is associated with REMS deficits

**Figure 5.**
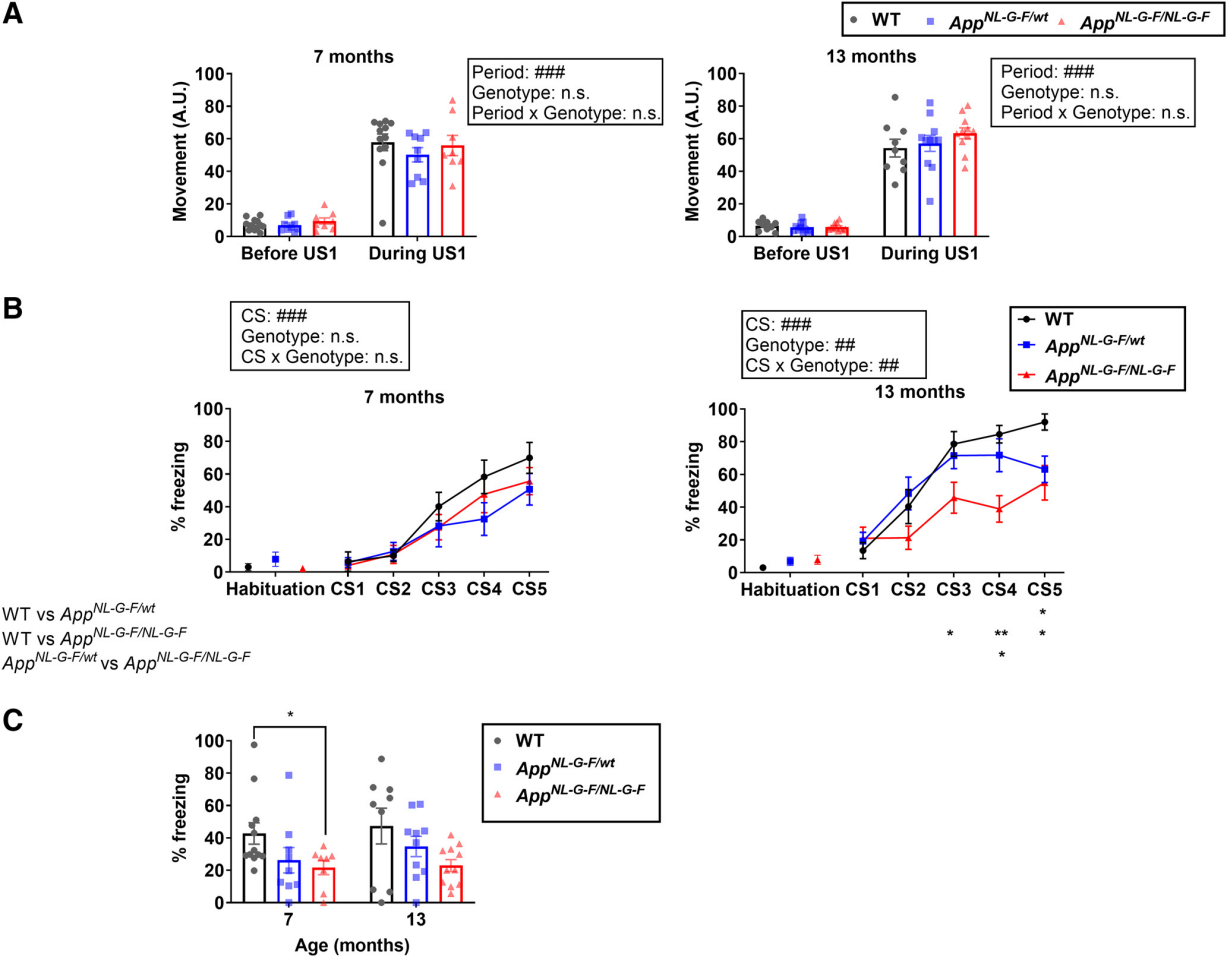
Impaired performance of *App^NL-G-F/NL-G-F^* mice in trace FC. ***A***, Movement of each seven-month-old and 13-month-old mouse before and during the first US; ###*p *<* *0.001, mixed ANOVA. ***B***, Percent time spent freezing during habituation or CS on day 1 (training). Each point represents mean ± SEM; ##*p *<* *0.01, ###*p *<* *0.001, mixed ANOVA; *p <* *0.05, **p <* *0.01, *post hoc* Games–Howell multiple comparison test. ***C***, Percent time spent freezing during CS on day 2 (retrieval). Bar graphs represent the mean ± SEM. Each point represents an individual mouse; *p <* *0.05, Games–Howell multiple comparison test. Detailed results of the statistical tests are described in Extended Data Figure 5-1.

Both sleep impairment and cognitive decline are commonly associated with the clinical stage of AD (Prinz et al., 1982; McCurry et al., 1999; Liguori et al., 2014). The *App^NL-G-F/NL-G-F^* mice exhibited various age-dependent sleep deficits, especially in REMS. Here, we addressed whether the detected REMS defects were associated with learning and memory impairments. To investigate the correlation between sleep parameters and learning and memory performance at the individual level, the mice used in the sleep study were also subjected to a learning task. The FC task is a commonly used memory task in which an aversive US (foot-shock) is associated with some CS, typically a visual or an auditory cue. *App^NL-G-F/NL-G-F^* mice are reported to perform normally in a contextual FC protocol, even at 15–18 months of age (Sakakibara et al., 2018). Here, we focused on trace FC. The trace FC is another hippocampus-dependent learning paradigm (McEchron et al., 1998; Quinn et al., 2002) that assesses temporal associative memory. In trace FC, a temporal gap is set between the CS (auditory tone) and the US (Huerta et al., 2000; Misane et al., 2005). Association of the temporally separated CS and US requires brain areas that are not essential for the delay FC (in which there is no gap between the CS and US), including the hippocampus and the prefrontal cortex (Gilmartin and Helmstetter, 2010). On day 1, the CS followed by the US was administered five times to mice at either seven or 13 months of age. Importantly, in all genotypes, the first US evoked a similar increase in movement, indicating that the sensitivity to the US itself was unaltered (Fig. 5*A*). At seven months, both the heterozygous and homozygous mice normally learned the association between the CS and US on day 1, as assessed by a gradual increase in the freezing rate during the CS (Fig. 5*B*). On day 2, the freezing rate following exposure to the tone was reduced in the homozygous mice compared with the WT control mice, suggesting impaired retention or recall of the memory (Fig. 5*C*). At 13 months, the homozygous mice, and to a lesser extent the heterozygous mice, exhibited learning impairment on day 1 compared with the WT control (Fig. 5*B*). The homozygous mice also exhibited a trend toward a reduction in the freezing rate on day 2 (Fig. 5*C*).

10.1523/ENEURO.0093-20.2020.f5-1 Extended Data Figure 5-1 Detailed results of the statistical analyses in Figure 5. Download Figure 5-1, XLSX file.

**Figure 6.**
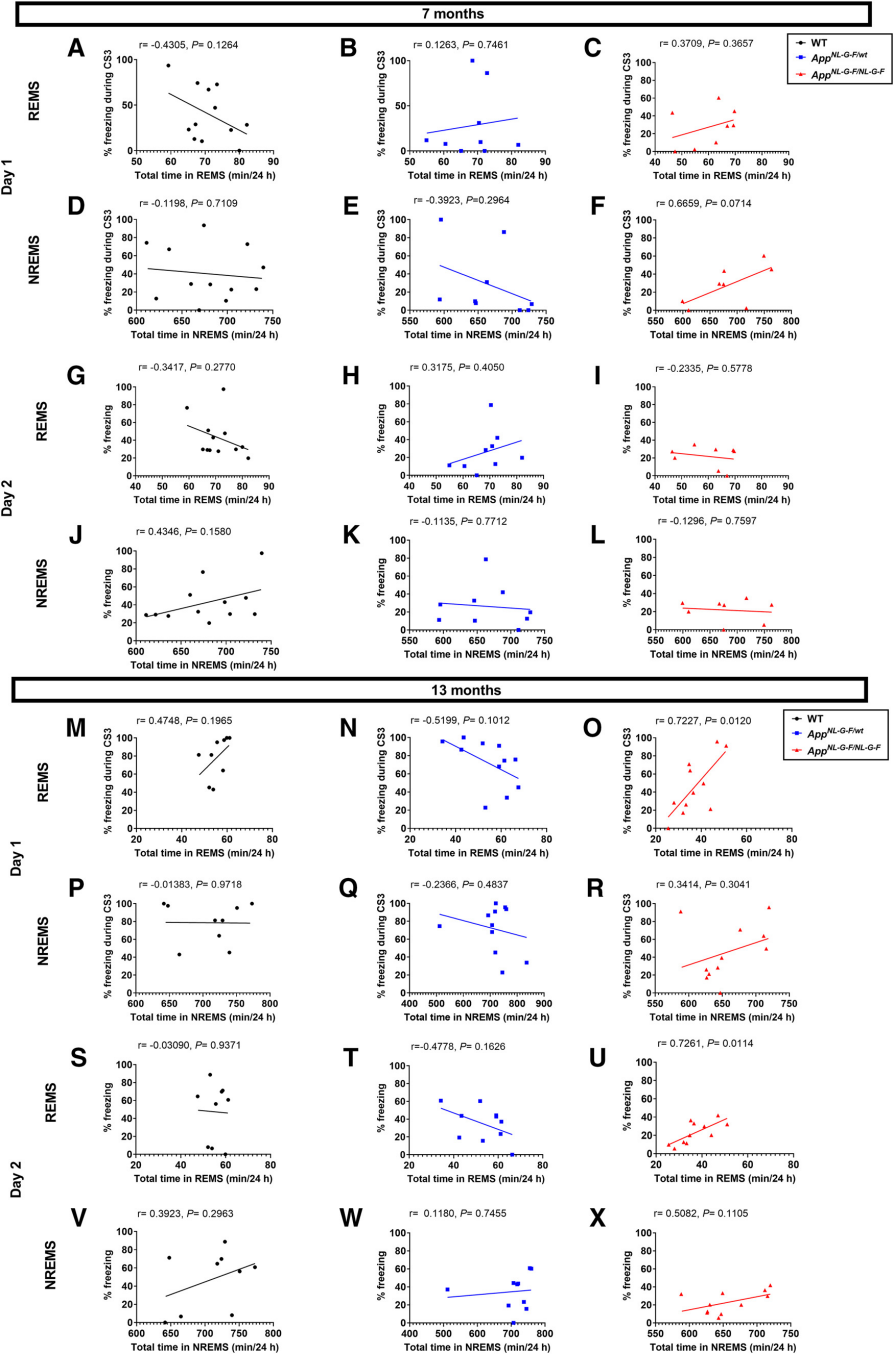
Correlations between the amount of time spent in each sleep stage and the performance in trace FC. ***A–L***, Correlation between the total amount of REMS or NREMS and the percent time spent freezing either during the third CS on day 1 (training) or during CS on day 2 (retrieval) in WT, *App^NL-G-F/wt^* and *App^NL-G-F/NL-G-F^* mice of the younger age. ***M–X***, Correlation between the total amount of REMS or NREMS and the percent time spent freezing either during the third CS on day 1 (training) or during CS on day 2 (retrieval) in WT, *App^NL-G-F/wt^* and *App^NL-G-F/NL-G-F^* mice of the older age. Each point represents an individual mouse. The line in each graph represents the regression line. Pearson’s correlation coefficient (*r*) and *p* value are provided.

To address whether the learning and memory deficits were associated with the sleep abnormalities, we performed correlation analyses between various sleep parameters and the freezing rate in the trace FC test (Fig. 6). The freezing rate during the third CS on day 1, which was reduced in the 13-month-old homozygous mice and had apparently not yet reached a plateau in the control WT mice, strongly and positively correlated with the total time in REMS (Fig. 6*O*). A similar trend was observed for the freezing rate on day 2 (Fig. 6*U*). By contrast, in the seven-month-old mice, although deficits in both REMS and trace FC were observed in the homozygous mice, no significant correlation was detected between the freezing rates and any sleep parameters tested (Fig. 6*C*,*F*,*I*,*L*), suggesting a specific correlation of the learning ability with REMS duration at the older age. We detected no significant correlation between the freezing rates and any sleep parameters tested in *App^NL-G-F/wt^* or WT mice (Fig. 6).

### Accumulation of Aβ in brain regions involved in REMS regulation

**Figure 7.**
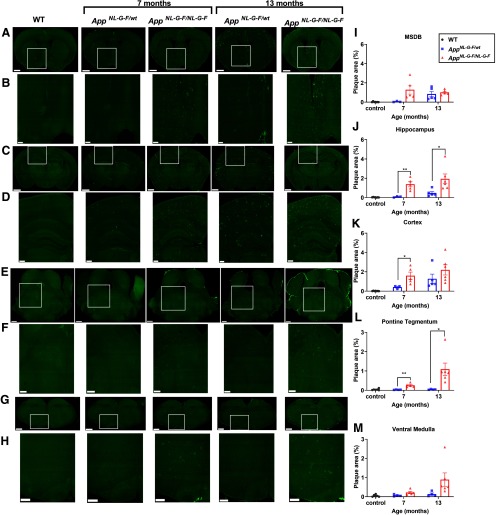
Aβ deposition in brain areas related to REMS regulation in *App^NL-G-F/wt^* and *App^NL-G-F/NL-G-F^* mice. ***A–H***, Representative images of brain sections immunostained for Aβ. Images in ***B***, ***D***, ***F***, ***H*** are higher magnifications of the areas enclosed in ***A***, ***C***, ***E***, ***G***. Sections contain the following brain regions: MSDB (***A***, ***B***), hippocampus and cortex (***C***, ***D***), pontine tegmental area (***E***, ***F***), and ventral medulla (***G***, ***H***). Scale bar: 2.5 mm (***A***, ***C***, ***E***, ***G***) and 1.0 mm (***B***, ***D***, ***F***, ***H***). ***I–M***, Quantification of Aβ plaque areas in MSDB (***I***), hippocampus (***J***), cortex (***K***), pontine tegmental area (***L***), and ventral medulla (***M***). Bar graphs represent the mean ± SEM. Each point represents an individual mouse; *p <* *0.05, **p <* *0.01, Welch’s *t* test. Detailed results of the statistical tests are described in Extended Data Figure 7-1.

Amyloidosis does not proceed in a uniform manner across all brain areas. One critical advantage of the AD mouse model used in this study is that the *App* expression is predicted to faithfully recapitulate the endogenous pattern (Sasaguri et al., 2017). Thus, we next addressed Aβ accumulation in brain areas related to REMS in these mice. First, consistent with previous reports (Saito et al., 2014; Whyte et al., 2018), Aβ accumulation in the hippocampus and cortex increased with age in both the homozygous and heterozygous mice (Fig. 7*C*,*D*,*J*,*K*). The major sleep defect detected in the current study occurred during REMS. Many studies describe a crucial role of the brainstem in REMS regulation, especially the pontine tegmental area and the ventral medulla (Sakai et al., 2001; Boissard et al., 2002; Lu et al., 2006; Hayashi et al., 2015; Weber et al., 2015).

10.1523/ENEURO.0093-20.2020.f7-1 Extended Data Figure 7-1 Detailed results of the statistical analyses in Figure 7. Download Figure 7-1, XLSX file.

Aβ seemed to accumulate in these brainstem areas in a manner different from that in the hippocampus and cortex. In these areas, Aβ accumulation largely increased from seven to 13 months in the homozygous mice, whereas it was hardly detectable in the heterozygous mice, a pattern that somewhat resembled the progression of the REMS impairment (Fig. 7*E*,*F*,*G*,*H*,*L*,*M*). The basal forebrain cholinergic neurons also contribute to sleep-wake regulation and are well known to be damaged in AD (Whitehouse et al., 1981, 1982; Lee et al., 2005; Ozen Irmak and de Lecea, 2014; Xu et al., 2015). Among these neurons, cholinergic neurons in the MSDB project to the hippocampus and are involved in oscillatory activity, neurogenesis, and learning and memory (Yoder and Pang, 2005; Hasselmo, 2006; Zhu et al., 2017). Aβ accumulation in the MSDB also appeared to progress in a manner different from that in the hippocampus or cortex, with comparable levels in the homozygous mice at seven and 13 months and in the heterozygous mice at 13 months (Fig. 7*A–D*,*I–K*).

## Discussion

This is the first study to describe the sleep abnormalities exhibited by *App^NL-G-F^* homozygous and heterozygous mice and the association of these sleep abnormalities with learning ability. Sleep is regulated by various brain areas and neuronal subtypes. Thus, addressing the association between sleep and AD using mouse models that overexpress or ectopically express APP or presenilin could complicate interpretations. Unlike previous studies in which the applied mouse models carried either multiple copies of *App* or *presenilin* or use heterologous promoters to express these genes, the present study used a mouse model in which mutated *App* was singly knocked into the original *App* locus. Indeed, homozygous mice faithfully recapitulated several aspects of the sleep abnormalities associated with preclinical or early AD. First, the amount of REMS was decreased from an early age, when no changes in the amount of wake or NREMS were detected. This is consistent with a recent prospective study in humans showing that the reduction in the total time spent in REMS, but not in NREMS, is associated with a higher risk for AD (Pase et al., 2017). Second, during REMS, slow oscillatory activity was increased while fast oscillatory activity was decreased. Again, studies of patients with early-stage AD or mild cognitive impairment report a similar shift in oscillatory activity during REMS (Prinz et al., 1992; Petit et al., 1993; Brayet et al., 2016). Thus, we believe the *App^NL-G-F^* knock-in mouse is highly useful for elucidating the mechanisms underlying sleep deficits in AD.

The sleep architecture in the homozygous mice at six months of age was characterized by a decrease in REMS. At 12 months of age, the reduction of REMS was further pronounced, and NREMS was also reduced. By contrast, the sleep architecture of the heterozygous mice appeared mostly normal, even at 12 months of age. This might be explained by the time course of Aβ accumulation in brain areas crucial for REM sleep regulation. Accumulating evidence supports an essential role of the pontine tegmental area and ventral medulla in regulating REMS (Sakai et al., 2001; Boissard et al., 2002; Lu et al., 2006; Hayashi et al., 2015; Weber et al., 2015). In these two areas, in contrast to the hippocampus or cortex, Aβ was almost undetectable in the heterozygous mice. On the other hand, in the homozygous mice, Aβ in these two areas increased with age, consistent with the progressive decrease in REMS. Therefore, damage to the brainstem might be critical for the development of sleep deficits in AD. Recent studies also point to the roles of these areas in regulating NREMS, which might account for the decrease in NREMS at later stages (Anaclet et al., 2014; Hayashi et al., 2015). The basal forebrain cholinergic neurons are commonly damaged in AD and are involved in sleep-wake regulation (Whitehouse et al., 1981, 1982; Lee et al., 2005; Ozen Irmak and de Lecea, 2014; Xu et al., 2015). The time course of the Aβ accumulation in the MSDB of the basal forebrain, which contains many cholinergic neurons projecting to the hippocampus, in both the heterozygous and homozygous mice appeared not to be strongly correlated with the progression in sleep impairment. In addition to the brainstem and MSDB, various brain areas, including the hypothalamus and midbrain, are involved in sleep regulation. Further studies are required to determine damage to which neurons largely contributes to the sleep deficits. While the reduction in REMS at the younger age in the homozygous mice was consistent with human studies on preclinical or early stages of AD (Prinz et al., 1982; Pase et al., 2017), the reduction in the ratio of deep NREMS (stage 3 or 4), which is especially prominent in the advanced stages of AD (Prinz et al., 1982), was not obvious in the homozygous mice. Even at the older age, the homozygous mice exhibited normal δ power, although the total amount of NREMS was reduced. Thus, the homozygous mice, although an excellent model for preclinical or early stages of AD, might not recapitulate the sleep impairments that emerge in the advanced stages of the disease. In addition, there are also reports that patients with mild cognitive impairment, part of which will likely develop AD, exhibit reduction in the time spent in both REMS and deep NREMS (Westerberg et al., 2012), suggesting that sleep impairments accompanying AD are not uniform. It would be interesting to evaluate the relation between sleep impairment and the accumulation of Aβ in the pontine tegmental area and ventral medulla in patients with various stages of AD in future studies.

According to the results of cortical EEG spectral analyses in AD patients or patients with mild cognitive impairment, alterations in the oscillatory activity during REMS are suggested to be more sensitive biological markers of the disease than alterations during wake (Petit et al., 1993; Brayet et al., 2016). In such patients, EEG slowing, i.e., the simultaneous occurrence of an increase in the power of the slow (e.g., δ) component and a decrease in the power of the fast (e.g., α or β) component of the EEG power spectrum during REM sleep was observed. The homozygous mice in our study appeared to well recapitulate the EEG slowing during REMS, which, to our knowledge, is the first report of this in an AD mouse model. By contrast, some other AD mouse models exhibit an apparently opposite phenotype, i.e., a decrease in δ or θ power and an increase in γ power (Zhang et al., 2005; Jyoti et al., 2010; Schneider et al., 2014; Colby-Milley et al., 2015; Kent et al., 2018). Cortical and hippocampal oscillatory activities are regulated by both local circuits and various subcortical areas, including the brainstem. The altered oscillatory activities again highlight the advantage of using a single *App* knock-in mouse, in which the endogenous expression pattern of APP is faithfully recapitulated. For example, ectopic expression or overexpression of APP might lead to impaired inhibitory neurotransmission, considering that secreted APP can act on GABA B receptors (Rice et al., 2019). Of note, in another study using *App^NL-G-F^* homozygous mice, local field potential measurements with tetrodes from the entorhinal cortex in awake behaving mice detected impaired γ-θ coupling as early as five months (Nakazono et al., 2017). Thus, in future studies, measurements of neural activity during REM sleep with similar devices and analyses may allow for the detection of impaired oscillations at an even earlier age.

According to recent studies, *App^NL-G-F^* homozygous and heterozygous mice exhibit fairly mild behavioral defects, consistent with the notion that these mice represent a preclinical or early stage of AD. For example, homozygous mice perform normally in the contextual FC test, a spatial hippocampal-dependent task, even at 15–18 months (Sakakibara et al., 2018). In the current study, using trace FC, which assesses temporal associative memory, we were able to detect a memory deficit in homozygous mice at seven months. At 13 months, homozygous mice, and to a lesser extent, heterozygous mice exhibited impaired learning. Interestingly, at seven months, there was no correlation between the amount of REMS and learning or memory, whereas at 13 months, there was a strong and positive correlation. Perhaps, the memory deficit and REMS impairment originally develop independently at younger ages, but in the course of disease progression, somehow REMS impairment contributes to worsening of the learning and memory deficit. Postlearning REMS is crucial for memory consolidation (Boyce et al., 2016). As the 13-month-old heterozygous and homozygous mice displayed learning impairments during training, however, it is unlikely that defects of the postlearning sleep are the major cause. Therefore, if the REMS impairment does contribute to learning and memory deficits, it might be that REMS is somehow involved in the daily maintenance of the brain areas related to learning. Whereas recent studies have begun to elucidate the roles of NREMS in brain maintenance, e.g., by enhancing clearance of metabolites or by downscaling synaptic excitability (Xie et al., 2013; Norimoto et al., 2018), the contribution of REMS is far less understood. Future studies should address the possibility that impairments in REMS affect brain maintenance and contribute to the progression of AD.

## Synthesis

Reviewing Editor: Bong-Kiun Kaang, Seoul National University Decisions are customarily a result of the Reviewing Editor and the peer reviewers coming together and discussing their recommendations until a consensus is reached. When revisions are invited, a fact-based synthesis statement explaining their decision and outlining what is needed to prepare a revision will be listed below. Note: If this manuscript was transferred from JNeurosci and a decision was made to accept the manuscript without peer review, a brief statement to this effect will instead be what is listed below.

I agree with the responses that were made by the author. The manuscript should be published as it is.
